# Prediction of Transplant-Free Survival through Albumin-Bilirubin Score in Primary Biliary Cholangitis

**DOI:** 10.3390/jcm8081258

**Published:** 2019-08-19

**Authors:** Koji Fujita, Takako Nomura, Asahiro Morishita, Tingting Shi, Kyoko Oura, Joji Tani, Hideki Kobara, Kunihiko Tsutsui, Takashi Himoto, Tsutomu Masaki

**Affiliations:** 1 Department of Gastroenterology and Neurology, Faculty of Medicine, Kagawa University, Miki 761-0701, Japan; 2 Department of Medical Technology, Kagawa Prefectural University of Health Sciences, Takamatsu 761-0123, Japan

**Keywords:** cirrhosis, liver transplantation, primary biliary cholangitis, survival

## Abstract

Albumin–bilirubin (ALBI) grade is defined using the ALBI score, which is calculated based on total serum bilirubin and albumin. This study aimed to evaluate the diagnostic ability of the ALBI score for determining hepatic fibrosis stage and transplant-free survival in primary biliary cholangitis (PBC) patients. A total of 181 Japanese patients with biopsy-proven or serologically diagnosed PBC were enrolled. The pathological stage was assessed using the Scheuer classification. The ALBI score differentiated fibrosis in stage 4 from that of 3 in the biopsy-proven cohort (*p* < 0.05). With an ALBI score cut-off value of −1.679, the sensitivity and specificity were 100% and 91.1%, respectively, with a likelihood ratio of 12.3 to differentiate stage 4 from stages 1–3. The ALBI score at the beginning of ursodeoxycholic acid (UDCA) prescription correlated with the two prognostic scores calculated after 1-year UDCA treatment. Kaplan–Meier analysis showed that the baseline ALBI score differentiated liver transplant-free survival (*p* < 0.05). The ALBI score presented a greater hazard ratio for transplant-free survival than aspartate aminotransferase-to-platelet ratio index (APRI) in Cox proportional hazard model. In conclusion, ALBI score indicates pathological stage in Japanese PBC patients and scores before UDCA prescription predict better liver transplant-free survival, which correlated well with the two major prognostic scores. The prognosis-predicting ability of the ALBI score might surpass that of APRI.

## 1. Introduction

Primary biliary cholangitis (PBC) is a chronic, progressive, and cholestatic disease of the liver [1,2]. The only definitive treatment is orthotopic liver transplantation for PBC when the disease resists ursodeoxycholic acid (UDCA) treatment [3]. UDCA treatment was established as the first-line therapy for PBC in the 1990s [4,5]. The survival rate of early-stage PBC patients who are treated with long-term UDCA is similar to that of the general population [6,7].

Recently, a liver fibrosis biomarker, the aspartate aminotransferase (AST)-to-platelet ratio index (APRI), was reported to predict patients’ prognosis in PBC [8,9]. Furthermore, two prognosis scores have been developed to predict the risk of disease progression resistant to UDCA treatment and liver transplant; these scores are the GLOBE score and UK-PBC risk score [10,11]. Both scores are based on blood exams after one year of UDCA therapy, with consideration to the therapeutic response of the disease to the therapy.

The albumin–bilirubin (ALBI) grade has been investigated to predict the prognosis of cirrhosis patients with or without hepatocellular carcinoma (HCC) [12]. The ALBI grade is defined using the ALBI score, which is calculated based on levels of bilirubin and albumin in the serum and is representative of liver function in cirrhosis patients. This study aimed to evaluate the diagnostic ability of ALBI score for determining hepatic fibrosis stage and transplant-free survival, comparing with GLOBE score, UK-PBC risk score and APRI in PBC patients.

## 2. Experimental Section

### 2.1. Ethics

This study was conducted in accordance with the ethical principles of the Declaration of Helsinki and was approved by the Institutional Review Board of Kagawa University, Faculty of Medicine (Heisei-30-151) [13]. Informed consent was obtained from all patients for analysis of clinical data. For patients who had died and had no relatives listed in their clinical records, we provided opt-out methods for the relatives of the dead participants by publishing a summary of this study on our university website [14,15].

### 2.2. Patients

A total of 181 Japanese patients with PBC who underwent percutaneous liver biopsy examinations or were serologically diagnosed in a clinical setting between 1 November 1987 and 31 December 2018 were enrolled in this retrospective study. Patients who had HCC at the time of PBC diagnosis were excluded.

### 2.3. Clinical Data

The following clinical data were extracted from the patients’ medical records: age, sex, platelet count, AST, alanine aminotransferase (ALT), alkaline phosphatase (ALP), total bilirubin (T-Bil), and serum albumin (Alb). T-Bil (mg/dL) was converted to T-Bil (µmol/L) according to the following equation: T-Bil (mg/dL) × 17.2. ALBI score was calculated based on a calculation from a previous report: Log 10 T-Bil (µmol/L) × 0.66 + Alb (g/L) × (−0.085) [12]. Serological diagnosis of PBC was performed using an anti-mitochondrial antibody and an anti-mitochondrial M2 antibody [16]. Data for the anti-centromere antibody was also extracted for patients who were followed up for one year or more [17]. Another antibody specific to the disease, anti-gp210 antibody, was not routinely evaluated in our hospital [18]. APRI was calculated using the following equation: 100 × (AST (U/L)/upper limit of normal AST values (U/L))/(Plt (×10^9^/L) [19]. In our hospital, 35 U/L was applied as the upper limit of the normal AST values.

### 2.4. Histopathological Analysis

The pathological stage of PBC was evaluated using the Scheuer classification (stage 1, florid duct lesion; stage 2, ductular proliferation; stage 3, scarring; and stage 4, cirrhosis) by an experienced pathologist who specialized in liver pathology [20,21].

### 2.5. Statistical Analyses

Continuous variables, presented as median and interquartile range, were analyzed using the Mann–Whitney *U* test or Spearman’s rank correlation coefficient. Data described as patient number were analyzed by Fisher’s exact test. Cut-off values in ROC analysis were determined using the Youden index. Kaplan–Meier curves were analyzed using the log-rank (Mantel-Cox) test. GLOBE score represents the transplant-free survival rate for 3, 5, 10 and 15 years, as GLOBE score 3, 5, 10 and 15, respectively [10]. The UK-PBC risk score provides the probability that patients under UDCA treatment develop liver failure requiring liver transplantation within 5, 10 or 15 years from diagnosis, as UK-PBC 5, 10, and 15, respectively [11]. The two scores were calculated based on blood exam data one year after UDCA prescription. The GLOBE score was calculated using a relevant online calculator (https://www.globalpbc.com/globe). The UK-PBC risk score was computed using the calculator available online (http://www.uk-pbc.com/resources/tools/riskcalculator). Another prognostic biomarker, a model for end-stage liver disease score (MELD score) was calculated in its original equation excluding serum sodium levels [22,23]. The statistical analyses above were performed using Graphpad Prism 6 (GraphPad Software, La Jolla, CA, USA). Cox proportional hazard model was analyzed using EZR (Saitama Medical Center, Jichi Medical University, Saitama, Japan), a graphical user interface for R software (The R Foundation for Statistical Computing, Vienna, Austria) [24,25]. *P* < 0.05 was considered statistically significant.

## 3. Results

### 3.1. Characteristics of the Patients

This study comprised 80 biopsy-proven patients and 101 serologically diagnosed patients (Table 1). In the biopsy-proven cohort, 34 patients were classified as stage 1, 21 patients classified as stage 2, 19 patients classified as stage 3, and six patients classified as stage 4, based on liver biopsy examinations. In the serologically diagnosed cohort, 41 patients were positive for anti-mitochondrial antibody, 90 patients were positive for anti-mitochondrial M2 antibody, and 20 patients were negative for both antibodies. All other clinical data are presented in Table 1.

### 3.2. Diagnostic Ability of Pathological Stage

**Figure 1:**
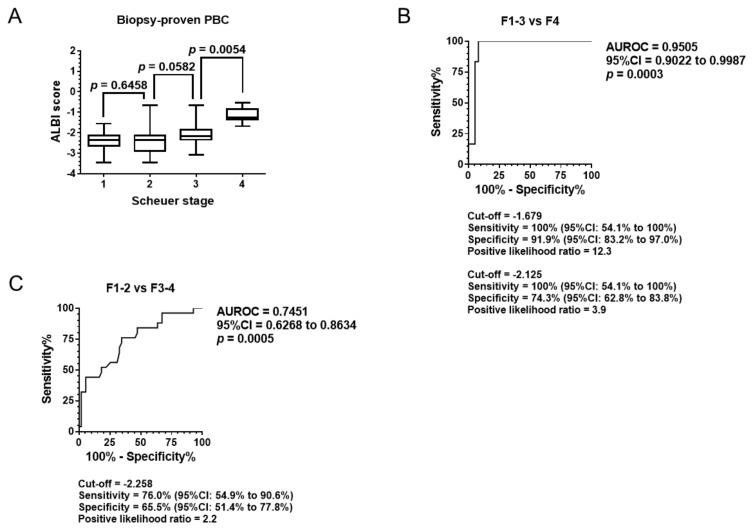
Diagnostic ability of pathological stage. (**A**) Relationship between Scheuer stage and the ALBI score in the biopsy-proven PBC cohort. (**B**) AUROC distinguishing cirrhosis from noncirrhotic status. (**C**) AUROC differentiating advanced fibrosis from nonadvanced fibrosis. ALBI, albumin–bilirubin; PBC, primary biliary cholangitis; AUROC, area under receiver operating characteristic; CI, confidence interval.

Differences in median values between two fibrosis stages were analyzed using the Mann–Whitney *U* test (Figure 1A). As a result, the ALBI score differentiated stage 4 from stage 3 (*p* < 0.05). The median value of the ALBI score for stage 3 presented tended to exceed the score for stage 2.

Receiver operating characteristic (ROC) analysis was performed to assess the ability of the ALBI score to distinguish cirrhosis (stage 4) from noncirrhotic status (stages 1–3) and advanced fibrosis (stages 3–4) from nonadvanced fibrosis (stages 1–2). As shown in Figure 1B, the area under the ROC (AUROC) curve distinguishing cirrhosis from noncirrhotic status was 0.9505. The AUROC differentiating advanced fibrosis from nonadvanced fibrosis was 0.7451.

With an ALBI score of −1.679 as a cut-off value, the sensitivity and specificity were 100% and 91.1%, respectively, and the positive likelihood ratio to differentiate cirrhosis from noncirrhotic status was 12.3 (Figure 1B). While the previous report presented the cut-off value of −2.125 to exclude cirrhosis in 382 patients with hepatitis C virus infection, the approximation in the current cohort was a cut-off value of −2.210, as shown in Supplementary Table 1 [26]. When the cut-off value = −2.210 was employed in Figure 1B, its sensitivity, specificity and positive likelihood ratio resulted in 100%, 74.32% and 3.895.

When a cut-off value of −2.258 was employed for differential diagnosis of advanced fibrosis from nonadvanced fibrosis, the sensitivity was 76.0% and the specificity was 65.5%, with a positive likelihood ratio of 2.2 (Figure 1C).

### 3.3. Baseline ALBI Score and Transplant-Free Survival

**Figure 2:**
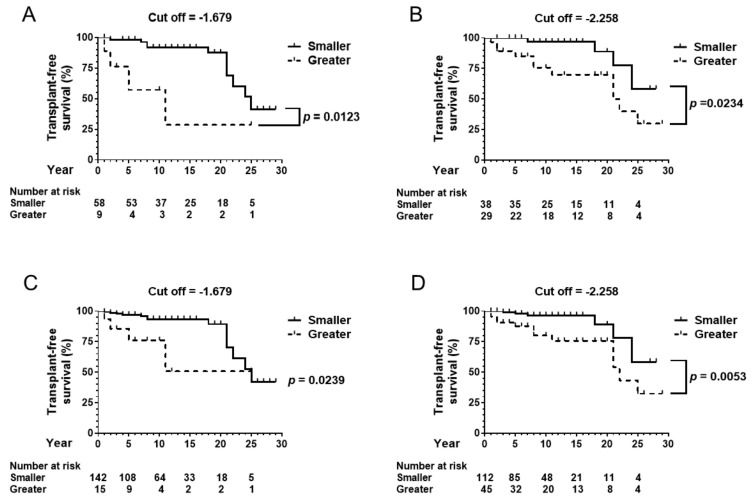
Transplant-free survival in the biopsy-proven cohort and the total cohort. (**A**) In the biopsy-proven cohort of 67 patients, the cut-off ALBI score of −1.679 differentiated the patients’ transplant-free survival. (**B**) The alternative cut-off value of −2.258 also divided the cohort by transplant-free survival. (**C**) In the total cohort of 157 patients who were followed up for one year or more, the cut-off ALBI score of −1.679 differentiated the patients’ transplant-free survival. (**D**) The alternative cut-off value of −2.258 also divided the cohort by transplant-free survival. ALBI, albumin–bilirubin.

In the biopsy-proven cohort, 67 patients were followed up for one year or more (Table 1). Fourteen patients died during the observation period. The observation of two patients was censored with liver transplantation. The cut-off ALBI score of −1.679 differentiated the patients’ transplant-free survival, as shown in Figure 2A (*p* < 0.05). The alternative cut-off value of −2.258 also divided the cohort based on transplant-free survival (Figure 2B, *p* < 0.05).

In the total cohort, 157 patients were followed up for one year or more (Table 1). Seventeen patients died, and two patients were transferred to other hospitals for liver transplantation during the observation period. The cut-off ALBI score of −1.679 differentiated the patients’ transplant-free survival in the total cohort, as shown in Figure 2C (*p* < 0.05). The alternative cut-off value of −2.258 also divided the cohort based on transplant-free survival (Figure 2D, *p* < 0.05).

### 3.4. Correlation of Baseline ALBI Score with GLOBE Score, UK-PBC Score, and MELD Score

**Figure 3:**
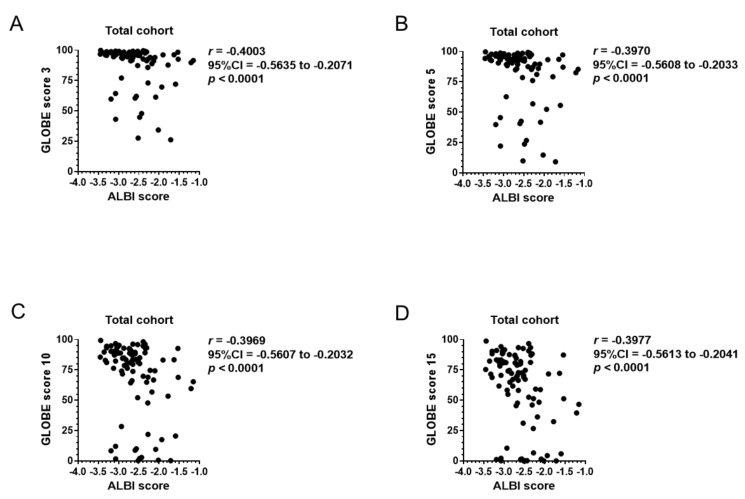
Correlation of the baseline ALBI score with the GLOBE score. (**A**–**D**) In 93 patients with available blood exam data one year after UDCA therapy, the ALBI score at the beginning of UDCA treatment significantly correlated with GLOBE scores 3, 5, 10 and 15. ALBI, albumin–bilirubin; CI, confidence interval; T-Bil, total bilirubin; Alb, albumin; Plt, platelet count; UDCA, ursodeoxycholic acid.

**Figure 4:**
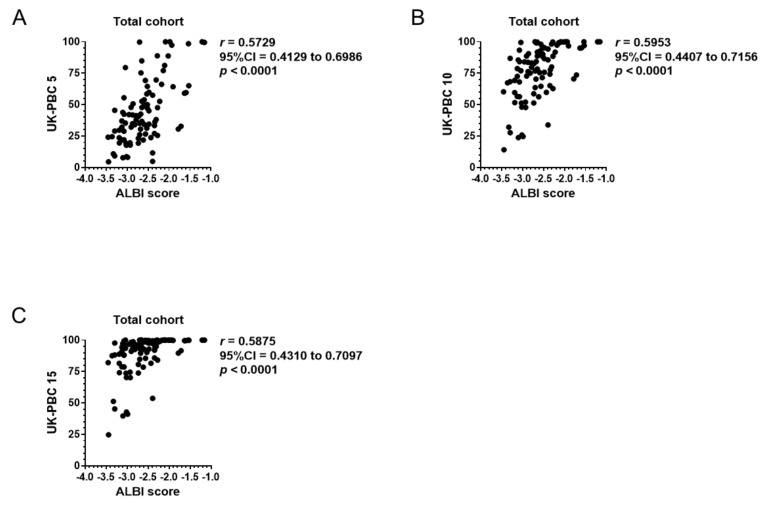
Correlation of the baseline ALBI score with UK-PBC score. (**A**–**C**) In 93 patients with available blood exam data one year after UDCA therapy, the ALBI score at the beginning of UDCA prescription significantly correlated with UK-PBC risk scores 5, 10 and 15. ALBI, albumin–bilirubin; CI, confidence interval; T-Bil, total bilirubin; Alb, albumin; Plt, platelet count; UDCA, ursodeoxycholic acid.

To assess the prognosis predicting values of the ALBI score before UDCA prescription, correlation of the baseline ALBI score with GLOBE and UK-PBC scores were computed for the total cohort. Among 139 patients who underwent UDCA therapy, blood exam data one year after UDCA prescription was preserved for a total of 93 patients (Table 2). As shown in Figure 3A–D, the ALBI score at the beginning of the UDCA prescription significantly correlated with GLOBE scores 3, 5, 10 and 15 after completion of one year of UDCA therapy. Similarly, UK-PBC risk score had a significant correlation with baseline ALBI score in UK-PBC 5, 10, and 15 (Figure 4A–C). However, ALBI score did not correlate to GLOBE or UK-PBC scores with a correlation coefficient larger than 0.7. The MELD score was calculated in 86 patients whose baseline PT-INR and serum creatinine were available. Among 86 patients, 80 patients presented a MELD score equal to or less than 8 with 1.9% of estimated 3-month mortality. The MELD score of the other six patients ranged between 9 and 16, suggesting 6% estimated 3-month mortality. Further analysis was not performed for the MELD score because the estimated 3-month mortality in the cohort was too small.

### 3.5. Transplant-Free Survival in Patients under UDCA Treatment

**Figure 5:**
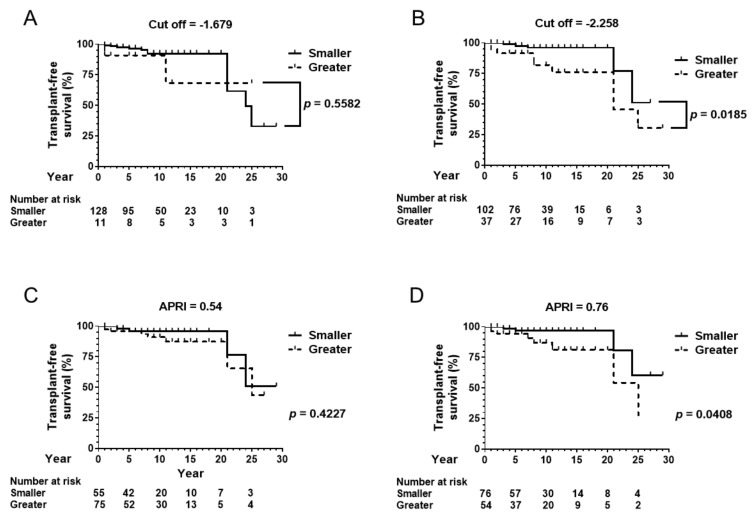
Transplant-free survival in patients under UDCA treatment. (**A**) In 139 patients who were prescribed with UDCA, the cut-off value for cirrhosis, an ALBI score of −1.679, did not differentiate transplant-free survival. (**B**) However, the cut-off value of −2.258, which is a threshold to differentiate advanced fibrosis from nonadvanced fibrosis, stratified transplant-free survival. In 139 patients who were prescribed with UDCA, baseline platelet count was available in 130 patients. (**C**) APRI = 0.54 did not differentiate transplant-free survival. (**D**) APRI = 0.76 significantly stratified the cohort by transplant-free survival. UDCA, ursodeoxycholic acid.

Among the 157 patients who were followed up for one year or more, 139 were prescribed with UDCA (Table 1). To validate prognosis predictive values of the ALBI score, the transplant-free survival of 139 patients was stratified using the cut-off values of −1.679 and −2.258. As shown in Figure 5A, the cut-off value for cirrhosis, an ALBI score of −1.679, did not differentiate transplant-free survival in the cohort. However, the cut-off value of −2.258, which was a threshold to differentiate advanced fibrosis from nonadvanced fibrosis, also differentiated transplant-free survival, suggesting that a smaller ALBI score predicts better transplant-free survival, even in the cohort under UDCA therapy (Figure 5B).

### 3.6. Prediction of Transplant-Free Survival by APRI

APRI, a conventional biomarker for liver fibrosis, serves as a prognostic biomarker in PBC. A cut-off value of APRI = 0.54 at baseline stratified risk of transplant-free survival [8]. Patients with baseline APRI greater than 0.76 experienced complications more frequently with cirrhosis-associated events than the others [9]. Among the 139 patients who were prescribed UDCA, baseline APRI was calculated for 130 patients. Though APRI = 0.54 did not differentiate transplant-free survival, patients with APRI less than 0.76 presented with significantly better transplant-free survival than the other patients (Figure 5C,D). Cox proportional hazard analysis was performed to evaluate proportional hazards of APRI smaller than 0.76 and an ALBI score smaller than −2.258. As shown in Table 3, an ALBI score of less than −2.258 yielded a significant risk reduction of transplant-free survival with a hazard ratio of 0.2669. APRI smaller than 0.76 also presented a tendency of better transplant-free survival with no statistical significance. When ALBI = −1.679 was applied as an alternative to ALBI = −2.258, neither ALBI score nor APRI presented hazard ratios with statistical significance.

## 4. Discussion

The following results were obtained in the present study. First, the ALBI score has the potential to diagnose the pathological stage in PBC cases. Second, a smaller ALBI score correlates with better transplant-free survival. Third, the ALBI score before UDCA therapy correlates with two major prognosis scores, the GLOBE and UK-PBC scores, which were calculated one year after completing UDCA therapy. Fourth, the prognosis-predicting ability of the ALBI score for transplant-free survival probably surpasses that of APRI’s.

The median values of the ALBI score differentiated stage 4 fibrosis from stage 3 (Figure 1A). Though the ALBI score was not significantly different between stages 2 and 3, ROC analysis revealed that the score was able to differentiate stages 3–4 from stages 1–2 (Figure 1C). The data suggests that the ALBI score has the potential to classify the PBC stage based solely on serum albumin and total bilirubin values.

Smaller ALBI scores at baseline predict better transplant-free survival in the biopsy-proven cohort (Figure 2A,B), in the total cohort (Figure 2C,D) and patients under UDCA therapy (Figure 5B), with each cohort including a large proportion of noncirrhotic patients. Originally, the ALBI score was established to classify only cirrhosis patients according to their prognosis [12]. A risk of post hepatectomy liver failure and long-term survival is stratified using the simple score [27]. It has a long-term impact on liver function on curative or locoregional therapy for hepatocellular carcinoma [28,29,30,31]. The present data also aligned with results from a previous study involving 63 Chinese PBC patients proving that a smaller ALBI score predicted improved event-free survival [32].

The two emerging prognosis scores, GLOBE score and UK-PBC score, focused on transplant-free survival based on the patients’ response to one year of UDCA therapy. While the ALBI score before UDCA therapy correlated with both scores, the correlation seemed to be greater with UK-PBC risk score than GLOBE score based on *r* values (Figure 4 and Figure 5). The greater correlation between ALBI score and UK-PBC score might be prevalent because these scores do not include age in their calculation. Though the correlation between the baseline ALBI score and GLOBE or UK-PBC score did not present stronger correlation with *r* values greater than 0.7, it might be partially attributed to the patients’ response to 1-year UDCA therapy [33].

The ALBI score is different from APRI in that the ALBI score does not include platelet count in its equation. While our data suggested that the ALBI score presented a more significant hazard ratio for transplant-free survival than APRI did, the ALBI score might provide another simple tool as an alternative to APRI in prognosis prediction of PBC.

This study has some limitations. First, a relatively small number of patients was included in the study. The cut-off ALBI score for cirrhosis in PBC patients was set at −1.690 in the current study. This cut-off value was greater than that in patients with Hepatitis C virus (HCV), which had a cut-off ALBI score of −2.125, as previously reported [26]. The discrepancy might be partially caused by the small number of patients with cirrhosis in the current cohort. The smaller number of cirrhotic patients might also explain why there is no significant difference in transplant-free survival when patients with UDCA therapy were divided based on cirrhotic status (Figure 5A). Recently, ALBI score = −1.60 was reported to stratify the cumulative survival rate of 325 patients with clinically diagnosed stable decompensated cirrhosis [34]. It remains to be investigated further whether clinically diagnosed cirrhosis should be evaluated similar to pathologically diagnosed cirrhosis. Second, conventional noninvasive biomarkers for liver fibrosis, Fibrosis-4 index and AST-to-platelet index (APRI), were not fully considered in this study because the biopsy-proven cohort included 53 of 80 patients whose baseline platelet count was available. While sufficient evidence has not yet been accumulated to determine cut-off values of the Fibrosis-4 index or APRI for cirrhosis in PBC, further evaluation should be performed [35].

## 5. Conclusions

The ALBI score indicates the pathological stage in Japanese PBC patients. Furthermore, a smaller ALBI score predicts better transplant-free survival before the commencement of UDCA therapy. The clinical application of the ALBI score has the potential to expand its application from cirrhosis to chronic hepatitis.

## Figures and Tables

**Table 1:** Patient baseline characteristics.

	Biopsy-Proven Cohort	Serologically Diagnosed Cohort	*p* Value	Total
Patient number	80	101	-	181
Follow up period < 1 year (*n*)	13	11	-	24
Follow up period > 1 year (*n*)	67	90	0.3780	157
UDCA therapy during the observation (*n*)	50	89	<0.0001	139
Blood exam data after one year of UDCA treatment (*n*)	15	78	<0.0001	93
Prescription of corticosteroid (*n*)	5	16	0.0607	21
Follow up period >1 year (year)	11 (7–20)	7 (4–11)	<0.0001	8 (4–14)
Age (year)	57 (51–65)	62 (55–72)	0.0014	60 (54–68)
Sex (Male/Female, *n*)	12/68	6/95	0.0490	18/163
Albumin (g/L)	36 (33–38)	40 (36–43)	<0.0001	38 (34–42)
AST (U/L)	57 (37–82)	38 (29–61)	0.0061	46 (31–71)
ALT (U/L)	49 (36–83)	35 (24–62)	0.0019	43 (26–73)
T-Bil (μmol/L)	13.8 (10.3–18.9)	10.3 (8.6–13.8)	0.0012	12.0 (8.6–15.5)
Platelet count (×10^9^/L)	*230 (154–275)	204 (159–245)	0.0790	^†^218 (159–249)
APRI	*0.868 (0.479–1.428)	0.562 (0.402–0.983)	0.0535	^†^0.648 (0.408–1.208)
Fibrosis stage 1/2/3/4 (*n*)	34/21/19/6	-	-	-
Positive for anti-mitochondrial Ab (*n*)	50	42	0.0069	92
Positive for anti-mitochondrial M2 Ab (*n*)	46	91	<0.0001	137
Negative for both Abs (*n*)	14	5	0.0074	19
Infection with HBV/HCV (*n*)	0/0	1/2	1.0000/0.5040	1/2
HCC incidence (*n*)	3	3	1.0000	6
Deaths or liver transplantation (*n*)	16	3	0.0003	19
Overall deaths (*n*)	14	3	0.0014	17
Liver-related deaths (HCC/Hepatic failure, *n*)	11 (3/8)	0	<0.0001 (0.0845/0.0012)	11 (3/8)
Liver transplantation (*n*)	2	0	0.1940	2

Continuous variables, presented as median and interquartile range, were analyzed using the Mann–Whitney *U* test. Data described as patient number were analyzed by Fisher’s exact test. *N*, patient number; UDCA, Ursodeoxycholic acid; Ab, antibody; HBV, hepatitis B virus; HCV, hepatitis C virus; HCC, hepatocellular carcinoma. * Data represent 53 patients in the biopsy-proven cohort. ^†^ Data represent 154 patients in the total cohort.

**Table 2:** Patient characteristics of those with available blood exams data after one year of UDCA therapy.

	Total
Patient number	93
Data at the beginning of UDCA therapy	
Albumin (g/L)	39 (36–43)
Creatinine (mg/dl)	0.59 (0.50–0.70)
T-Bil (μmol/L)	10.3 (8.6–13.8)
PT/INR	0.97 (0.93–1.04)
Platelet count (×10^9^/L)	214 (161–242)
Data after one year of UDCA therapy	
Albumin (g/L)	41 (39–43)
ALT (U/L)	23 (15–34)
ALP (U/L)	302 (239–418)
T-Bil (μmol/L)	10.3 (6.9–12.0)
Platelet count (×10^9^/L)	206 (156–254)
Positive for anti-centromere Ab (*n*)	30

Continuous variables were presented as median and interquartile range. *N*, patient number; UDCA, Ursodeoxycholic acid; PT/INR, prothrombin time/international normalized ratio; T-Bil, total bilirubin; Ab, antibody.

**Table 3:** Cox proportional hazard ratio analysis for transplant-free survival.

	Hazard Ratio	95% Confidence Interval	*p* Value
ALBI score < −2.258	0.2669	0.0716–0.9947	0.0491
APRI < 0.76	0.5807	0.1755–1.9220	0.3734

APRI, AST-to-platelet ratio index.
